# Modulation of chemokines in the tumor microenvironment enhances oncolytic virotherapy for colorectal cancer

**DOI:** 10.18632/oncotarget.7907

**Published:** 2016-03-04

**Authors:** Lily Francis, Zong Sheng Guo, Zuqiang Liu, Roshni Ravindranathan, Julie A. Urban, Magesh Sathaiah, Deepa Magge, Pawel Kalinski, David L. Bartlett

**Affiliations:** ^1^ University of Pittsburgh Cancer Institute, Pittsburgh, PA, USA; ^2^ Department of Surgery, University of Pittsburgh School of Medicine, Pittsburgh, PA, USA; ^3^ Department of Immunology, University of Pittsburgh School of Medicine, Pittsburgh, PA, USA; ^4^ Department of Bioengineering, University of Pittsburgh School of Medicine, Pittsburgh, PA, USA

**Keywords:** oncolytic virus, immunotherapy, tumor microenvironment, chemokine, pharmaceutical modulation

## Abstract

An oncolytic poxvirus such as vvDD-CXCL11 can generate potent systemic antitumor immunity as well as targeted oncolysis, yet the antitumor effect is limited probably due to limited homing to and suppressed activity of tumor-specific adaptive immune cells in the tumor microenvironment (TME). We reasoned that a chemokine modulating (CKM) drug cocktail, consisting of IFN-α, poly I:C, and a COX-2 inhibitor, may skew the chemokine (CK) and cytokine profile into a favorable one in the TME, and this pharmaceutical modulation would enhance both the trafficking into and function of antitumor immune cells in the TME, thus increasing therapeutic efficacy of the oncolytic virus. In this study we show for the first time *in vivo* that the CKM modulates the CK microenvironment but it does not modulate antitumor immunity by itself in a MC38 colon cancer model. Sequential treatment with the virus and then CKM results in the upregulation of Th1-attracting CKs and reduction of T*reg*-attracting CKs (CCL22 and CXCL12), concurrent with enhanced trafficking of tumor-specific CD8^+^ T cells and NK cells into the TME, thus resulting in the most significant antitumor activity and long term survival of tumor-bearing mice. This novel combined regimen, with the oncolytic virus (vvDD-CXCL11) inducing direct oncolysis and eliciting potent antitumor immunity, and the CKM inducing a favorable chemokine profile in the TME that promotes the trafficking and function of antitumor Tc1/Th1 and NK cells, may have great utility for oncolytic immunotherapy for cancer.

## INTRODUCTION

Oncolytic virotherapy (OVT) has demonstrated significant promise in both preclinical studies and clinical trials [1], and correlative studies confirm that OVT is a new form of immunotherapy for cancer [2–4]. In the last few years, more and more research groups have devoted their attention to vaccinia virus (VV) as an oncolytic virus (OV). This has been attributed to the unique properties of VV, especially its native tumor tropism, efficient cell-cell spread and high levels of transgene expression in tumor cells. The most advanced clinical developments are from Pexa-Vec (JX-594), a Wyeth strain oncolytic VV, which has minimal therapy-associated toxicities and demonstrated objective clinical responses in human cancer patients [5, 6]. We have previously demonstrated that the WR strain of oncolytic vaccinia virus vvDD, with dual deletions of viral genes encoding thymidine kinase (*tk*) and vaccinia growth factor (*vgf*), is a tumor-selective replicating and potent OV in animal models [7]. The virus is safe in non-human primates and human patients [8, 9]. However, further improvements are needed in order for the oncolytic VV to be highly efficacious. Investigators have worked to improve the efficacy of the virus in different ways, such as arming it with another immunostimulatory genes [2, 3, 10], such as cytokine (e.g. CD40L; IL-10) [11, 12], chemokine (e.g. CCL5) [13]. Combination strategies may further enhance both the efficacy and safety of this oncolytic virus [14, 15].

Previous studies have shown that the number of infiltrated CD8^+^ T cells is a good prognostic factor in human colorectal cancer [16–18], while a high density of tumor-infiltrating FOXP3^+^ T*reg* cells is associated with poor outcome in a number of solid cancer types, including ovarian [19], pancreatic [20], and hepatocellular carcinomas [21, 22]. One major factor affecting the infiltration of immune cells into the tumor niche is the CK profile and concentrations in the TME, and the expression of corresponding CK receptors on the surface of the immune cells [23, 24]. Therefore, trafficking to and accumulation of antitumor CD8^+^ T cells in the tumor tissue, would be expected to enhance the therapeutic efficacy of immunotherapy including OVT.

In order to enhance the trafficking of antitumor Tc1/Th1 cells into the TME, expression of Tc1/Th1-attracting CKs from a tumor-selective OV cells is a reasonable strategy. It has been known that CKs play important roles in cancer, such as intracellular signaling and intercellular communication in the TME, tumor cell migration and invasion, as well as trafficking of immune cells [23, 24]. CXCL11, also called I-TAC, a ligand for receptors CXCR3 and CXCR7, attracts CD8^+^ T cells and NK cells to the tumor or other inflamed sites [25–27]. It has potent antitumor activity *in vivo* involving attraction of CD8^+^ T lymphocytes [26]. Fc-fused CXCL11 could function as a strong adjuvant to enhance antigen-specific CD8^+^ T cell responses [28]. CXCL11 can also bind to a different variant of CXCR3 receptor and mediate inhibition of endothelial cells and thus tumor angiogenesis [29]. We have constructed an oncolytic vaccinia virus expressing murine CXCL11, vvDD-CXCL11 [30].

The multifaceted TME influences viral infection, replication, and propagation within the tumor, as well as antiviral and antitumor immune responses, and thus has a huge impact on OVT [31–35]. Cancer cells infected by an oncolytic poxvirus undergo programmed necrosis and apoptosis with release of ATP, HMGB1 as well as immunogenic endoplasmic reticulum chaperone gp96, all danger signals to the innate immune system, making it a type of immunogenic cell death (ICD) [36–39]. This ICD together with tumor-associated antigens (TAAs) released from dying cancer cells may prime for a potentially potent antitumor immunity [2]. The next challenge is to control T cell trafficking to the tumor sites [40]. In addition to virally-directed expression of Tc1/Th1-attracting CKs, we have recently found that a CK modulation (CKM) drug cocktail including IFN-α, poly I:C, and a cyclooxygenase (COX)-2 inhibitor, could enhance the production of Tc1/Th1-attracting CKs such as CCL5 and CXCL10, greatly reduce the production of CCL22, a CK associated with infiltration of regulatory T cells (T*reg*) in human tumor tissue explants *in vitro* [41]. If this effect could be reproduced in a tumor-bearing host *in vivo*, the application of the drugs may be a great way to modulate the TME and promote the trafficking of tumor-specific effector T cells and NK cells into the tumor tissues, thus enhancing the therapeutic efficacy.

We reasoned that OV would induce an acute inflammatory signal in the TME, prime DCs with danger signal along with TAAs and activate tumor antigen-specific adaptive CD4^+^ and CD8^+^ T cells in the draining lymph nodes and in circulation; while CKM would modulate the CK microenvironment leading to increased immunostimulatory CKs such as CCL5 and CXCL10, and concurrent reduction of immunosuppressive CKs (CCL22 and CXCL12). These effects would promote the trafficking of antitumor Th1/T*eff* cells into tumors and retain their functions in the TME, thus enhancing the overall immunotherapeutic efficacy. In the current study, we present data to support this hypothesis. Our results demonstrate that this rational combination of a potent oncolytic virus (vvDD-CXCL11) with a drug cocktail for tumor-selective reprogramming of the CK profile in the TME enhanced the oncolytic immunotherapy in a model of syngeneic colorectal cancer.

## RESULTS

### Increased tumor growth and decreased survival in CXCR3-knockout mice

**Figure 1:**
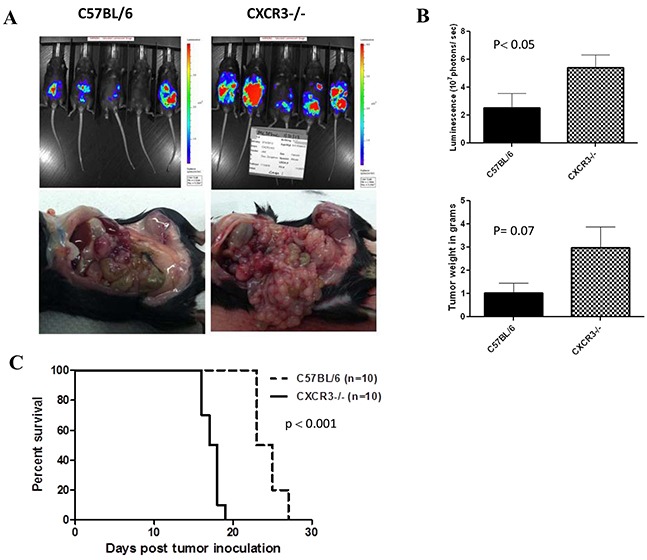
Tumor growth in CXCR3 knockout and wild type C57BL/6 mice **A.** Mice were injected i.p. with 5.0E5 MC38-luc cells on day 0. Whole animal live optical imaging was performed on various days as described in Materials and methods. Shown are images on day 15 (above panel), representative picture of tumor nodules in the peritoneal cavity (lower panel). **B.** The difference of tumor sizes was reflected in both the tumor luminescence (p< 0.05) and tumor burden (p= 0.07). **C.** The survival of tumor-bearing CXCR3 KO and wild type (WT) mice are plotted demonstrating a significant difference in length of survival (p < 0.001).

CXCR3 is expressed primarily on activated T lymphocytes and NK cells [42]. CXCR3 binds to the CXC subclass of CKs including CXCL11, as well as CXCL9, CXCL10, and CXCL4 (for CXCR3-B only) [29, 43]. These CKs play important roles in the trafficking of activated T and NK cells into tumor tissues. Therefore we wondered if tumor growth of MC38 colon cancer would be affected in CXCR3 knockout mice. MC38-luc colorectal cancer grew faster in CXCR3−/− mice than the wild type mice, as shown by parameters in both optical light imaging and real tumor burden (weight) (Figure 1; panels A and B). Not surprisingly, MC38-luc tumor-bearing CXCR3−/− mice lived shorter than those of wild type mice (Figure 1C). These results suggested that one or more ligands for CXCR3 are important to the inhibition of tumor growth, most likely for their functions in promoting trafficking to tumors and activating T and NK cells in the tumor tissue.

### Construction and initial characterization of the new vvDD-CXCL11

**Figure 2:**
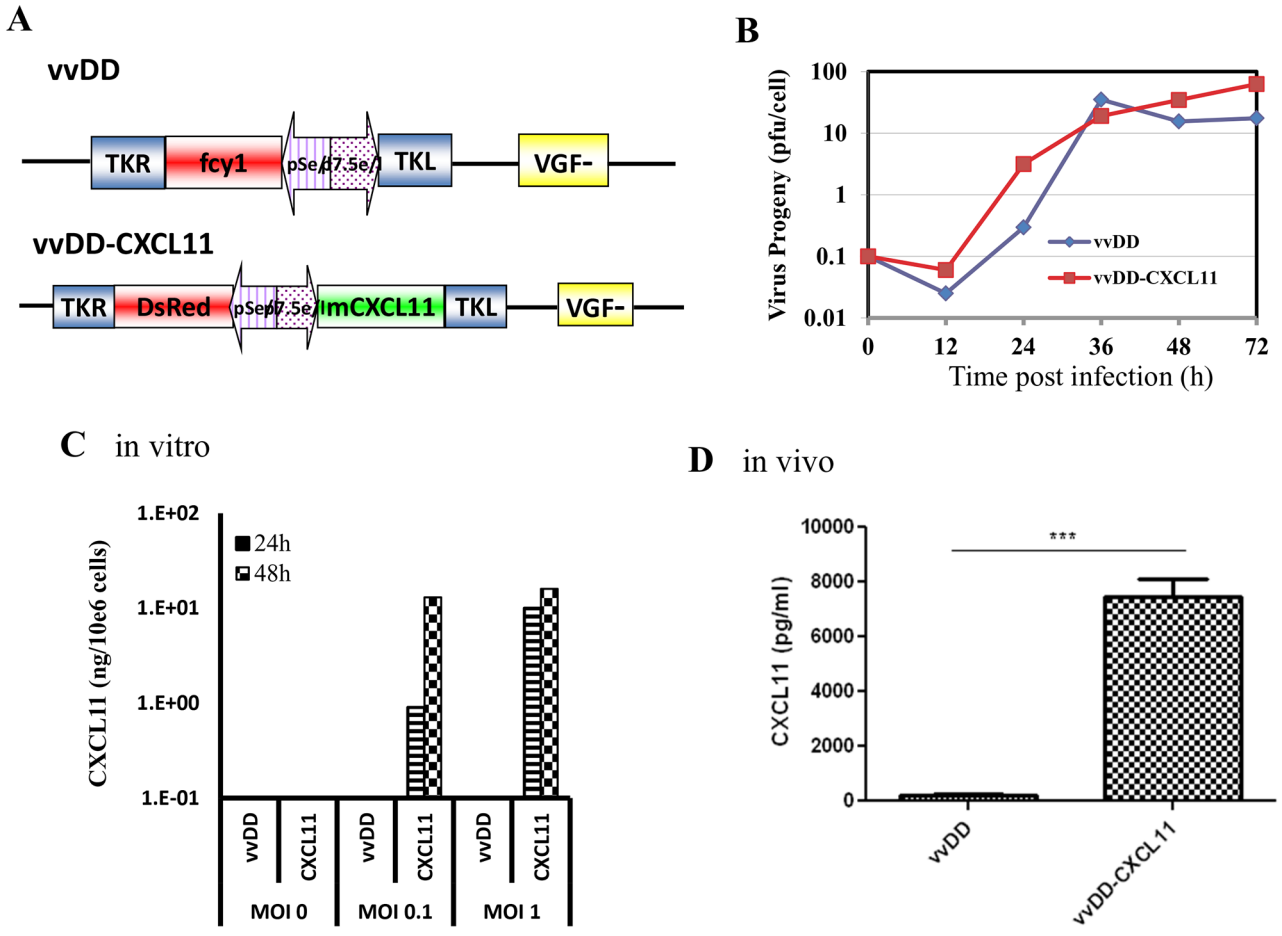
The new virus vvCXCL11 was functional as an oncolytic virus and expressed CXCL11 from infected cancer cells *in vitro* and *in vivo* **A.** Schematic representation of the new virus vvCXCL11 and parental virus vvDD (full name vvDD-CD). **B.** Viral replication in MC38 cancer cells. **C.** Expression and secretion of CXCL11 from infected MC38 cancer cells. CXCL11 in the conditioned medium was quantified by an ELISA assay. **D.** The levels of CXCL11 in tumor tissues were quantified by making the tumor tissue lysate and measured via ELISA assay. P value: ***, p < 0.001.

Thus, we were interested in overexpressing one of the CXCR3 ligands in the tumor tissues, which could attract CXCR3-expressing Tc1/Th1 and NK cells from circulation into the TME and subsequently activate them. We picked CXCL11 and made a new oncolytic VV expressing murine CXCL11, as described in Materials and Methods (Figure 2A). The new virus replicates as well as the parental virus in MC38 colon cancer cells (Figure 2B). The high levels of CXCL11 secreted from infected MC38 cancer cells were determined *in vitro* and in MC38 tumors *in vivo* as determined by ELISA assays (Figure 2C and 2D). These results demonstrated that vvDD-CXCL11 is a replicating oncolytic virus and it secrets functional CXCL11 from infected cancer cells *in vitro* and *in vivo*.

### CXCL11 expression from the virus increases recruitment of CD8^+^ T cells, but has little effect on tumor growth

**Figure 3:**
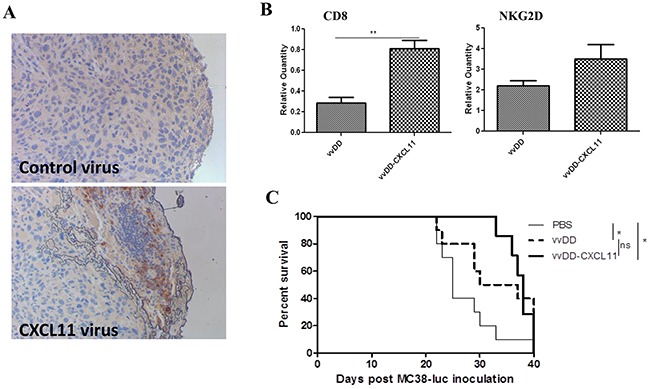
CXCL11 expressed from the virus enhances infiltration of CD8^+^ T Cells into the tumor tissues, but yields no significant survival benefit **A.** IHC of CD8^+^ cells in the tumor tissues. The control virus is vvDD and CXCL11 virus is vvDD-CXCL11. **B.** Real-time RT-PCR for mRNAs encoding CD8 (marker for CD8^+^ T cells and CD8^+^ DC) and NKG2D (activation marker on NK and T cells). Two days after virus treatment, tumor tissues were harvested and the total RNA was purified from the tissues. **C.** Kaplan-Meier plot for survival of tumor-bearing mice treated with PBS, vvDD, or vvDD-CXCL11. The p values are indicated: *p <0.05; **p < 0.01; ns = not significant.

We then tested the effects of the armed virus in comparison to the parental virus on the trafficking of CD8^+^ T cells and NK cells, as well as the inhibition of tumor growth and animal survival in tumor-bearing mice. We observed significantly increased infiltration of CD8^+^ T cells in tumors from mice treated with vvDD-CXCL11, as shown by both IHC (Figure 3A) of the tumor tissues and real-time RT-PCR on total RNA extracted from the tumor tissues for markers CD8 and NKG2D (Figure 3B). Surprisingly, the increased infiltration of CD8^+^ T cells did not translate into better therapeutic efficacy as animals treated with either virus survived with similar kinetics, even though the mice treated with either virus survived longer than those treated with PBS saline (Figure 3C).

### CKM modulates the immunological TME yet little effect on host survival

**Figure 4:**
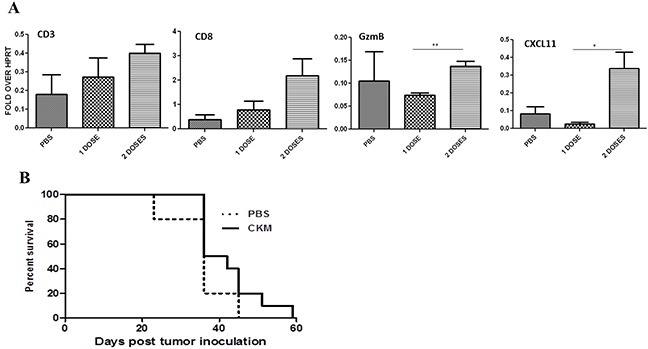
Two doses of CKM enhanced CD3 CD8^+^ markers and CXCL11, but not granzyme B levels in the tumor tissue, and did not enhance the survival of MC38-luc tumor-bearing C57BL/6 mice **A.** Shown are qRT-PCR results on the levels of mRNAs for CD3, CD8, granzyme B (GzmB), and CXCL11 from tumor tissue after treatment with PBS, 1 or 2 doses of CKM. Tumor tissues were harvested at 48 h post final treatment and purified total RNA was used for RT-qPCR. p = ns, not significant; * p< 0.05; **p< 0.01. **B.** Kaplan-Meier analysis of survival of mice bearing MC38-luc tumor treated with PBS or CKM for a total of 6 doses. p = ns.

We have previously shown that CKM cocktail can modulate the immunological properties of the TME using a tumor tissue explant culture system [41]. This includes enhanced production of CKs CCL5 and CXCL10, and reduced production of CCL22/MDC, a CK associated with infiltration of T*reg*. In this study, we have now applied this CKM cocktail in a tumor model *in vivo* for the first time. As a preliminary experiment, we examined intratumoral mRNA levels for CD3, CD8, Granzyme B, and CXCL11 after treatment with PBS, 1 dose of CKM, or 2 doses of CKM using Real-time RT-PCR analyses. CD3, CD8, and CXCL11 mRNA levels were increased after 2 doses of CKM compared to PBS. Granzyme B was increased with two doses of CKM compared to one dose (Figure 4A). Despite these encouraging indicators, 6 doses of CKM treatment alone did not show any benefit in survival of the animals (Figure 4B).

### The combination of vvDD-CXCL11 and CKM modulates the TME, recruits tumor-specific CD8^+^ T cells and NK cells, and enhances antitumor efficacy

**Figure 5:**
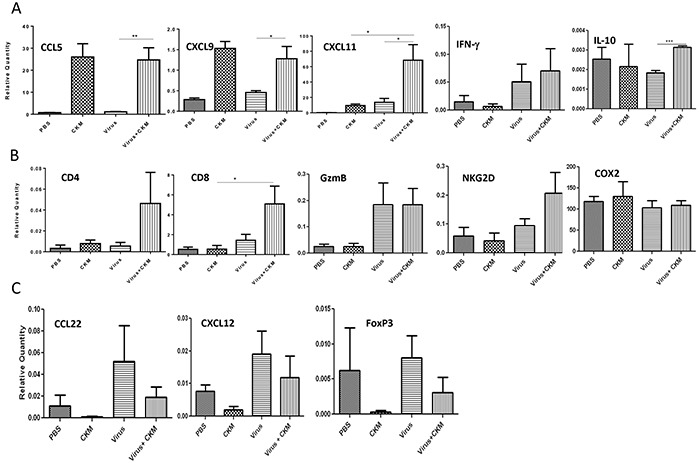
CKM modulates the TME into one with an immunostimulatory CK profile, enhances the trafficking of CD4 and CD8 T cells and potentiates killer activity **A.** Real-time PCR analyses on levels of mRNAs for cytokine and CK markers in the TME. Tumor tissues were harvested at 48 h post final treatment and purified total RNA was used for RT-qPCR. Included are pro-inflammatory CKs (CCL5, CXCL9 and CXCL11), and cytokines IFN-γ and IL-10. **B.** Immune cell markers CD4, CD8, killer cell activation marker granzyme B (GzmB) and NKG2D, as well as the mRNA level for COX-2. **C.** T*reg* cell-attracting CKs (CCL22 and CXCL12) and T*reg* cell marker FoxP3. RT-qPCR was performed as described in Materials and Methods. The mRNA levels were then expressed as relative quantity in relative to the house-keeping gene HPRT (Fold over HPRT). Data are representatives from two independent experiments. The virus used is vvDD-CXCL11 and labeled just as “virus” for simplicity. Standard symbols are used to indicate p values: *p < 0.05; **p < 0.01.

We then explored the potential of the combination regimen on the TME, especially the profile of the CKs, recruitment of immune cells, and antitumor potency in comparison to either monotherapy alone. First we analyzed the profiles of five important immunological markers, three CKs and two cytokines (Figure 5A). For CCL5, CXCL9 and IFN-γ, the virus vvDD-CXCL11 alone induced their expression while CKM did not. CKM did induce a moderate level of CXCL11 expression, while the armed virus expressed high levels of CXCL11 from the transgene in the virus as expected. The dual treatment maintained high levels of CCL5 and CXCL9 as in CKM treatment, a high level of IFN-γ as in virus treatment, and elevated CXCL11 expression. Thus, dual treatment led to equal or higher levels of expression of inflammatory CKs and IFN-γ. Interestingly, virus slightly inhibited the production of IL-10, but addition of CKM restored the IL-10 production to the base level.

We then examined the cellular markers in responses to either monotherapy or dual therapy (Figure 5B). There was little change of CD4 marker in the tumor tissue in responses to either monotherapy. However, dual treatment showed a trend of increased CD4^+^ cells (due to small number of animals studied, p value was insignificant). As for CD8 expression, an increase was seen in the virus treatment group, but not with CKM treatment. Dual treatment led to significant enhancement of CD8^+^ cells (p < 0.05). For the activating marker granzyme B (GzmB) and NKG2D (for both T and NK cells), virus treatment and dual treatment, but not CKM alone, tended to increase expression of both markers. This might indicate that the combination led to not only enhanced infiltration of CD8^+^ T and/or NK cells, but also activated state as both activating makers were expressed at higher levels. As a control, COX-2 mRNA has not been changed with either mono- or dual treatments, but this is expected as celecoxib in the CKM is for the inhibition of COX-2 enzymatic function, not transcription of the gene. While validation using cell sorting of tumor infiltrating leucocytes would confirm these results, it was not feasible due to the small size of the peritoneal tumors at this time point.

We also examined the effects on tumor-promoting markers related to T*reg* cells, including CCL22 [44], CXCL12 [45] and FoxP3 (Figure 5C). The virus tended to induce the immunosuppressive CCL22 and CXCL12 and had no much effect on FoxP3, the cellular marker for T*reg* cells. However, the CKM cocktail tended to inhibit expression of CCL22, CXXL12, as well as FoxP3 in our *in vivo* tumor model. In the dual treatment, the addition of CKM reduced the virus-induced expression of CCL22 and FoxP3. We have also seen a trend towards reduction of virus-induced CXCL12 by the treatment with CKM.

In summary, the addition of the CKM to the virus treatment promoted the production of pro-inflammatory cytokines such as IFN-γ, CKs such as CCL5, CXCL9 and CXCL11, enhanced the numbers of CD4^+^ and CD8^+^ immune cells and the immune cell activation marker (NKG2D), while inhibiting the production of immunosuppressive CKs (CCL22, CXCL12) and the infiltration of T*reg* cells. These effects may lead to potent induction and function of antitumor immunity in the TME.

### vvDD-CXCL11 induces potent systemic anti-tumor immunity and the dual therapy produces enhanced therapeutic efficacy

**Figure 6:**
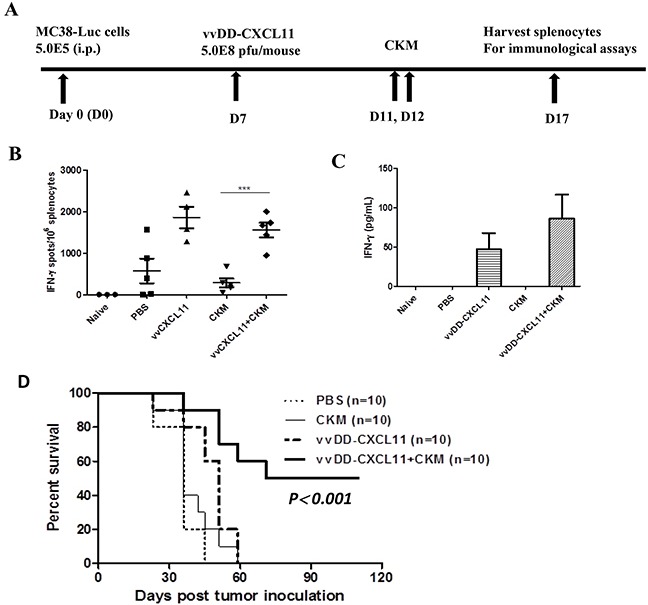
The virus vvCXCL11, but not CKM, has major effects on the systemic immunity, and dual treatment synergistically prolongs the survival of MC38-luc tumor-bearing mice **A.** Scheme of the treatments of mice and immunological assays. **B.** IFN-γ ELISPOT assay on the splenocytes collected on day 5 after final treatment. p <0.001 (***) when comparing CKM to the virus or virus+CKM; p = ns when comparing virus to virus+ CKM. **C.** Total amounts of secreted IFN-γ from cocultures of splenocytes with irradiated MC38-luc cells incubated for 48 hr. **D.** Kaplan-Meier survival analysis of MC38-luc carcinomatosis-bearing C57BL/6 mice treated with PBS, CKM, vvDD-CXCL11, or the combination (n=10 for each group). The median survival are, 28 days for PBS or CKM, 48 days for vvDD-CXCL11, at least 115 days for the combination (p<0.001 between singular versus the combination treatments).

We examined the systemic antitumor immunity on splenocytes isolated from the mice treated with PBS, the virus alone, CKM alone or the combination under the treatment procedure as shown (Figure 6A). IFN-γ ELISPOT assay (Figure 6B) showed that there were very few IFN-γ producing tumor-specific cells (mainly CD8^+^ T cells, CD4^+^ T cells and NK cells) in control mice treated with PBS saline. Virus-treated mice produced increased tumor-specific IFN-γ producing immune cells, while PBS or CKM treatment led to little, if any, production of tumor-specific IFN-γ-producing immune cells. The combination treatment of CKM and the virus led to a high level of production of tumor-specific IFN-γ^+^ immune cells, comparable to those treated with the virus alone. We have also performed an ELISA assay to quantify the amounts of IFN-γ in the supernatants of the co-cultures of splenocytes and irradiated MC38 cancer cells (Figure 6C). The results showed that splenocytes from naïve mice, PBS or CKM-treated mice secreted very low levels of IFN-γ while the splenocytes from mice treated with virus alone or the combination secreted much higher levels of IFN-γ (Figure 6C). Thus, both ELISPOT and ELISA assays indicated the same pattern of immunological activation. These results, together with the previous data, strongly suggest that the CXCL11-expressing oncolytic virus elicited potent systemic antitumor immunity, while the CKM targeted the tumor immunological microenvironment, promoting the trafficking of antitumor immune cells into the tumor and maintaining their antitumor activity, with little direct effect on the initial step of activation of antitumor immune cells.

Finally, we examined the effects of the combination therapy on tumor growth and survival (Figure 6D). In the MC38-luc-bearing mice, PBS control therapy was associated with a median survival of 34 days. CKM treatment alone did not benefit the tumor-bearing mice. Mice treated with vvDD-CXCL11 survived longer (median survival 48 days), demonstrating the therapeutic effect of the virus. The most dramatic effect on survival was associated with the combination treatment, where the median survival extended to at least 115 days.

## DISCUSSION

It has previously been demonstrated that oncolytic poxvirus induced infected cancer cells into programmed necrosis and apoptosis with release of ATP, HMGB1 as well as immunogenic endoplasmic reticulum chaperone gp96, all danger signals to the innate immune system [36–39] and key signals for induction of antitumor immunity [2, 46, 47]. We have previously shown the improved results of oncolytic vaccinia viruses armed with the chemokines CCL5 or CCL19 compared to the parent VV [13]. While those armed viruses were more effective than the parental virus, they also demonstrated improved results when combined with an alpha dendritic cells-mediated cancer vaccine or adoptive transfer of CIK cells. In a recent manuscript, we identified vvDD-CXCL11 as having the unique property of inducing high levels of autologous tumor-reactive splenocytes in an immunogenic tumor (AB12 mesothelioma) and functioning in the induction phase of the immune response [30]. Because of the increased systemic anti-tumor immunity, we felt it would be ideal to combine this effect in a non-immunogenic tumor with a regimen that modifies the tumor microenvironment and enhances effector cell trafficking.

In the current study, we have moved one step forward and demonstrated that vvDD-CXCL11 exerted oncolysis, elicited potent systemic adaptive antitumor immunity, and attracted CD8+ T-cells into the TME. However, we have also shown that this anticancer activity by itself is not enough for high therapeutic efficacy. Our data suggests that this is explained by the observation that the immunosuppressive TME inhibited the cytotoxic function of the infiltrated immune cells and that the virus may induce immunosuppressive factors.

A number of studies have shown that the modulation of the immunological TME is critical in immunotherapy regimens. As discussed previously, our prior work demonstrated that a drug cocktail consisting of IFN-α, poly I:C, and Celebrex (a cyclooxygenase (COX)-2 inhibitor), could enhance the production of immunostimulatory CKs (such as CCL5 and CXCL10), and reduce the production of immunosuppressive CK CCL22 (T*reg* attractant) in human tumor tissue explants *in vitro* [41]. All three drugs were required for uniform effects. We applied this cocktail to the *in vivo* tumor model for the first time here and showed that CKM indeed modulated the TME into a favorable microenvironment for antitumor immunity *in vivo*. The combinatorial regimen generated a favorable cytokine and chemokine profile, as indicated by higher levels of IFNγ, IL-10, CCL5, CXCL9 and CXCL11, and reduced levels of CCL22 and CXCL12. As a result, we observed higher levels of NKG2D (an activation marker for T and NK cells), granzyme B (a cytotoxicity marker for T and NK cells), and a lower level of FOXP3 (a marker for T*reg* cells). Further analyses indicated that the virus itself elicited a higher level of systemic anti-tumor vaccination, as indicated by increased tumor specific IFN-γ secreting immune cells (T and NK cells), while CKM did not. These combined effects led to potent antitumor activity and significantly prolonged survival in MC38-tumor bearing mice.

It is important to point out that the immunogenicity of the tumor model would greatly affect the outcome with the combination regimen. We have recently studied vvDD-CXCL11 in the murine AB12 mesothelioma model which is highly immunogenic [48]. In that model, the oncolytic virus alone, without CKM, was quite efficacious [30]. It is easy to speculate that the immunologic TME is more favorable for elicitation of antitumor immunity in an immunogenic tumor (such as AB12 mesothelioma), and thus CKM treatment would become functionally redundant and thus play marginal effect in further improving the oncolytic therapeutic efficacy. Given that most solid human tumors are non-immunogenic, the combination of vvDD-CXCL11 and CKM may provide a mechanism to impact both the induction and effector phases of anti-tumor immunity and produce an effective anti-tumor immune response. However, more models need to be examined before we can provide a solid conclusion on this hypothesis.

In summary, we have rationally developed a combination strategy to produce a highly efficacious therapeutic regimen to treat a colorectal carcinomatosis model in syngeneic mice. The oncolytic virus exerts its intrinsic capacity of oncolysis, CXCL11 expression leads to systemic adaptive antitumor immunity and attraction of cytotoxic T-cells, while the CKM cocktail modulates the TME to one immunologically favorable with CK profile skewing toward attracting Th1/Tc1 cells, enhancing the attraction of activated Tc1/Th1 cells and NK cells, and sustained activity of attracted immune cells to destroy cancer cells in the TME. This novel combinatorial regimen may be efficacious for treating non-immunogenic human tumors in late stages of disease.

## MATERIALS AND METHODS

### Cell lines

The cell lines ofhuman cervical cancer HeLa, monkey kidney fibroblast CV1,MC38 and firefly luciferase gene-tagged MC38 (MC38-luc) colon cancer have been previously used in the laboratory [49]. All these cell lines were grown in Dulbecco's modified Eagle's medium (DMEM) supplemented with 5-10% heat-inactivated fetal bovine serum (FBS; Atlanta Biologicals, Lawrenceville, GA), L-glutamine, and penicillin/streptomycin (Invitrogen, Carlsbad, CA). Cell lines were maintained in an incubator at 37°C with 5% CO_2_.

### Recombinant VV

WR strain derived recombinant viruses have been used in this study. vSC20, the virus with mutation in the vgf loci, was used to infect CV-1 cells, which were then transfected with a *tk* shuttle vector containing the expression cassette for murine CXCL11. The recombinant virus vvDD-CXCL11 was selected by DsRed expression and flow sorting and multiple rounds of plaque purification. After three rounds of plaque purification in 96-well plates, viral DNA from individual plaque was extracted and the inserted sequence was confirming by DNA sequencing. The control virus vvDD-CD (with yeast fcy1 gene) has been previously described [50].

### Viral replication assays *in vitro*

Viral replication assays were performed as previously described [37, 51]. Briefly, 1.0 × 10^5^ MC38-luc cells, in 6-well plates were infected with vvDD or vvDD-CXCL11 at MOIs of 0.1, 1.0 or 10 in 1 ml of 2% FBS-containing-Dulbecco's Modified Eagle Medium (DMEM) for 2 h at 37°C. Following infection, cells were cultured in DMEM-10% FBS until harvesting at 12, 24, 48 and 72 h after viral infection. The cell pellets were homogenized using a Precellys 24 Tissue Homogenizer/Grinder (Bertin Technologies, Rockville, MD), to release virions. The infectious viruses in the resulting cell lysates were titered on CV-1 cells, and expressed as plaque forming unit (pfu) per mL.

### Mice, MC38 peritoneal carcinomatosis (PC) model, live animal imaging, treatments and survival monitoring

CXCR3-knock-out mice (B6.129P2-Cxcr3tm1Dgen/J) were obtained from the Jackson Laboratory (Bar Harbor, ME). Female C57BL/6 mice, about 6 weeks old, were obtained from Taconic Biosciences, Inc. (Germantown, NY). All animal studies were approved by the Institutional Animal Care and Use Committee at the University.

The MC38-luc PC model was established by injection of 5.0 × 10^5^ MC38-luc cells i.p. into C57BL/6 mice. Tumor establishment and progression was monitored by live animal IVIS imaging right before the injection of the virus and various days afterwards. The *in vivo* optical imaging in living animals was performed using a Xenogen IVIS 200 Optical *In Vivo* Imaging System (Caliper Life Sciences, Hopkinton, MA, USA), with technical assistance from the Small Animal Imaging Core Facility.

The viruses (at 5.0E8 pfu/mouse/0.5 mL) were given i.p. on day 7 post tumor cell inoculation. The CK-modulating (CKM) drug cocktail was made freshly. Each mouse was injected i.p. with 200 uL of the CKM solution (containing 10,000 U of IFN-α, 50 mg of polyI:C, and 0.075 mg of Celecoxib). Unless indicated otherwise, the injection was taken place on days 11, 12, 15, 16, 19, and 20 (injection for two days and rest for two days). IFN-α was obtained from PBL Biomedical Labs (Piscataway, NJ), poly I:C from Sigma (St Louis, MO) and celecoxib from Biovision Inc (San Francisco, CA).

The health and survival of treated and mock-treated mice was closely monitored. All mice subjected to peritoneal tumors were monitored via caliper measurements for changes in abdominal girth. There are two criteria for death of animals: natural death due to the disease or any animal in which abdominal girth exceeded 1.5x the original measurement was euthanized and recorded as a death [51].

### ELISA assay for quantification of murine CXCL11

The concentration of CXCL11 in the supernatants of cancer cell culture or tissue lysate of tumor tissues was quantified using mouse CXCL11 Duoset ELISA kit according to the manufacturer's instructions (R & D Systems, Minneapolis, MN).

### Mouse interferon-γ ELISPOT assays

The assays were performed essentially as described [52]. Briefly, plates were coated with 100 mL of 15 mg/ml of anti-mouse IFN-γ mAb (clone AN18, Mabtech Inc., Cincinnati, OH). Splenocytes (5.0E5 cells/well) were restimulated with γ-irradiated (10,000 rad) MC38-luc cells (1.0E5 cells/well) for 24 h. After appropriate washes, 100 mL of 1.0 mg/mL biotinylated secondary antibody (clone R4-6A2-biotin, Mabtech, Inc.) in 0.5% FBS-containing PBS was added and incubated at room temperature for 2 h. The spots were developed by using Vecstatin Elite ABC kit (Vector Laboratories, Inc., Burlingame, CA).

### TaqMan qPCR analysis of mRNA expression

Unless specified otherwise, for *in vivo* experiments, mice were sacrificed and tumor tissues were harvested for purification of total RNA at 48 h after final treatment. Tumor cells or biopsied tissues were placed in Lysing Matrix D Tubes (MP Biologicals) containing RLT buffer from RNeasy kit (Qiagen, Valencia, CA), and agitated using a FP120 homogenizer (MP Biologicals, Santa Ana, CA). After spin supernatants from the lysis matrix tubes were transferred into new tubes. The total RNA was extracted using the RNeasy kit. One microgram of total RNA was used for cDNA synthesis, and 25 to 50 ng of cDNA was used for TaqMan PCR analysis for levels of individual mRNAs on the StepOnePlus™ Real-Time PCR system (Applied Biosystems, Inc., Thermo Fisher Scientific, Waltham, MA), as we have described previously [41]. The TaqMan^®^ Gene Expression Assays for individual genes including primers were obtained from the Applied Biosystems. The house-keeping gene hypoxanthine guanine phosphoribosyltransferase (HPRT) was used as a reference gene for the mRNA levels of genes of interest.

### Immunohistochemistry

At day 12 post tumor cell implantation and 2 days after virus treatment, tumor tissues were harvested. The tissues were fixed in 4% paraformaldehyde and embedded in paraffin. 10 micrometer sections were cut using a microtome, and placed on slides. CD8 immunohistochemistry was performed following the established protocols using the Vectastain ABC kit (Vector Laboratories, Burlingame, CA). CD8 primary antibody was used at 1:50 dilution, and biotinylated anti rat secondary antibody at 1:100 dilution.

### Statistical analysis

Raw data were recorded electronically and statistical analyses were performed with SPSS Statistics Software version 18 (IBM, NY, USA), or Prism (GraphPad Software, Inc., La Jolla, CA). An alpha value (p) of 0.05 was considered as statistically significant, and all p-values were two-sided. The standard symbols are used in the figures. * indicates p < 0.05; **p< 0.01; ***p <0.001; and ns = not significant.
